# Analysis of smile aesthetics using the SmileCurves digital template

**DOI:** 10.1590/2177-6709.25.1.080-088.sar

**Published:** 2020

**Authors:** Carlos Alexandre Câmara

**Affiliations:** 1 Universidade Estadual do Rio de Janeiro (Rio de Janeiro/RJ, Brasil).

**Keywords:** Aesthetics, Smile, Orthodontics, Dental aesthetics, Template

## Abstract

**Introduction:**

The aesthetic analysis of a smile may be facilitated by the use of a template that provides several dental aesthetic references and support for the diagnosis, simplifying it and defining guidelines for the aesthetic planning of orthodontic and integrated treatments.

**Objective:**

To describe a simple and objective procedure for the evaluation of smile aesthetics using the SmileCurves digital template (SCT), based on the superimposition of intraoral photographic images and close-up views of a smile.

**Conclusion:**

SCT is a simple and objective tool for the aesthetic analysis of a smile.

## RESUMO

**Introdução:**

A análise da estética do sorriso pode ser facilitada pelo uso de um *template* que agregue diversas referências estéticas dentárias e que auxilie e simplifique o diagnóstico, oferecendo diretrizes para o planejamento estético de tratamentos ortodônticos e integrados.

**Objetivo:**

Apresentar uma metodologia que possibilite a avaliação estética do sorriso, de maneira simples e objetiva, através do *template* digital SmileCurves (TSC), utilizado por meio da sobreposição de imagens de fotografias intrabucais e do sorriso aproximado.

**Conclusão:**

O uso do *template* TSC facilita a análise da estética do sorriso de modo simples e objetivo.

## INTRODUCTION

As a rule, aesthetic smile evaluations are subjective for most individuals. However, although this subjective analysis of smile agreeability may be possible, several references may objectively support the evaluation of correct aesthetic and occlusal positioning of anterior maxillary teeth. Requisites range from the analysis of ideal proportions of tooth width and height to the positioning of the long axis of teeth (angles and inclinations), as well as the associations between the white (teeth), pink (gingiva) and black (outlines) aesthetics.

Several types of analyses have been developed to evaluate this large number of variables.^1^ ^,^ ^2^ ^,^ ^3^ All have the main purpose of providing parameters and references to work as guidelines for the correct positioning of the maxillary anterior teeth.

Diverse norms, references and parameters should be adopted to evaluate aesthetic demands and, simultaneously, to define guidelines to reposition teeth and achieve ideal aesthetic outcomes.

In face of the plethora of concepts and analyses available, dentists should be able to find objective and simple forms of evaluation. Although the knowledge of all these variables is useful, it is necessary to understand them clearly, so that the assessment of fundamental aspects does not take a long time.

Following the concepts of simplicity and practicability, without compromising accuracy and usefulness, the use of templates responds to planning demands when the purpose is to evaluate aesthetic needs accurately.

Concepts that are already well known and validated make it possible to design and use reference models that include several parameters, thus optimizing and simplifying evaluations. For that purpose, the SmileCurves template was designed by using the Diagrams of Dental Aesthetic References (DDAR),^1^ together with the Six Horizontal Smile Lines.^2^ These two analytical frameworks alone include a number of variables, enough to build a guide for the correct positioning of anterior maxillary teeth in a beautiful smile.

## DIAGRAM OF DENTAL AESTHETIC REFERENCES (DDAR)^1^ (FIG. 1)

The Diagram of Dental Aesthetic References (DDAR) follows a rule, the dominance of maxillary central incisors (MCI), and six characteristics: symmetry (Fig. 1.1), tooth axes (Fig. 1.2), gingival contour (Fig. 1.3), interproximal contacts (Fig. 1.4), incisal edges (Fig. 1.5) and dental proportions (Fig. 1.6).

**Figure 1:**
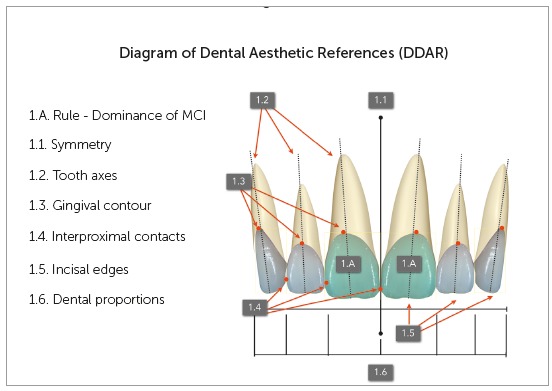
Diagram of Dental Aesthetic References (DDAR).

The purpose of these diagrams is to provide accurate information about positions and proportions of teeth in relation to each other, as well as their relation to gingiva and the lips. DDAR is organized in six boxes, which include the maxillary incisors and canines, and their borders are specific for each aesthetic reference. Each box includes its tooth, respecting its limits, as well as the rule of dominance and the reference of the MCI boxes.^1^

Mathematical relationships - such as the golden proportion, Plato's theory of beauty, the rule of Polykleitos, the diagonal of the square (Albers), and Lysippus' proportion - have been suggested for the determination of mesiodistal spaces.^4^ ^,^ ^5^ ^,^ ^6^ Although the golden proportion is the most popular and most frequently used, not all patients have this proportion.^6^ ^,^ ^7^ In these cases, the dental diagram should be used to treat each one individually. That is, the outcome should be a harmonious relationship, reflected in the visibility of anterior teeth. The frontal view shows that teeth should be visualized in a decreasing order, beginning with central incisors (dominance). In other words, DDAR does not use standard measurements, but, rather, the subjective perception of agreeability of proportions, supported by the objectivity of the size of boxes. One of the reasons why numbers were not used in DDAR was the possibility of using it with young patients, whose immature gingival development and the absence of dental wear result in teeth whose clinical crown width/height are different from those of more mature patients, whose width/height is about 65-80%. Even sets of teeth that do not have the so-called ideal proportions of height and width, as in young patients, may have aesthetically agreeable proportions when they follow a regressive proportion or graduation, together with dominance of maxillary central incisors.

## SIX HORIZONTAL SMILE LINES^2^

The horizontal smile lines complement and complete the information provided by DDAR, which facilitate the visualization and understanding of the white (teeth) and pink (gingiva) aesthetics, together with the participation of the lips. In the context of informative tracings, the six horizontal smile lines are determined and efficient visualizations of the correct or incorrect positioning of the important structures that are part of the smile, which facilitates its interpretation and the understanding of the meaning of its framework. 

### Six Horizontal Smile Lines (Fig. 2):

**Figure 2:**
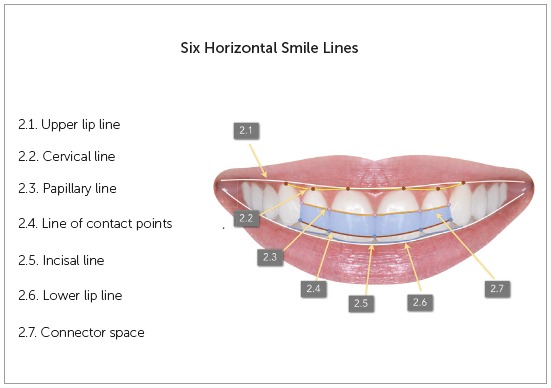
Six Horizontal Smile Lines.

1. Upper lip line;
2. Cervical (gingival) line;
3. Papillary line;
4. Line of contact points;
5. Incisal line;
6. Lower lip line.

### 1. Upper lip line (Fig. 2.1)

The upper lip line, represented by the lower edge of the lip, may be classified as high, medium or low, according to the degree of possible exposure of anterior and posterior teeth. 

### 2. Cervical line (Fig. 2.2)

The cervical, or gingival, line is formed by the union of the zenith of maxillary canines, lateral incisors and central incisors. The zenith, the most apical point of the gingival contour, of the maxillary teeth is usually distal to the tooth long axis. However, this rule does not always apply to maxillary lateral incisors. The gingival limit of these teeth may be centered on the long axis. As the zeniths of the maxillary canines are often higher than those of the lateral incisors and about the same height of the central incisors, the cervical line appears as a convex line in relation to the occlusal plane. This would be the ideal form of the cervical line.

### 3. Papillary line (Fig. 2.3)

The papillary line is formed by the tips of the gingival papillae located between maxillary canines and lateral incisors, and between maxillary lateral and central incisors. No studies have evaluated their standard height. In other words, these is no definition of an ideal model of relationship between papillae heights. However, based on studies that evaluated the ideal height of central incisors and the relationship of the height of the papilla tips and the position and size of teeth,^8^ ^,^ ^9^ an ideal line should be parallel to the line formed by the contact points.

### 4. Line of contact points (Fig. 2.4)

The interproximal contacts of maxillary anterior teeth decreases from the canines. The contact between canine and lateral incisor is higher than the contact between the lateral and central incisors; the contact between central incisors is even lower. These contact points should be tight, unless there is a discrepancy in the mesiodistal diameter of the crown.^2^ The position of the interproximal contact is associated with tooth position and morphology.^10^ Therefore, the line that unites these points should be parallel to the incisal line when there are no discrepancies in size, shape and angle of the anterior teeth.

### 5. Incisal line (Fig. 2.5)

The incisal line follows the edges of the maxillary anterior teeth. A frontal view of young patients should ideally show the incisal edges of the central incisors positioned below the edges of the lateral incisors and canines. In this distribution, the incisal line resembles the outline of a "soup plate". Depending on the relationship of the edges of the central and lateral incisors and canines, this outline may resemble a flat plate (canines, central and lateral incisors leveled) or an inverted soup plate (canines lower than central and lateral incisors).

### 6. Lower lip line (Fig. 2.6)

The relationship between the curve of the incisal edge of the maxillary anterior teeth and the curve of the upper edge of the lower lip should be harmonious during voluntary smile.^11^ This relationship of the incisal edges of maxillary canines and incisors with the lower lip is called smile arc.^12^ ^,^ ^13^ The curve of the incisal edges should ideally be parallel to the lower lip, and the incisal edges should be a little distant from or slightly touching the lip. However, this is only possible when the lower lip forms a natural curve, with the corners of the mouth turned upwards, and when the incisal edges follow this curve. In other words, for an agreeable effect, dental and labial structures must be symmetrical. If lips or teeth limit the parallel relationship to each other, the smile arc will not be possible. Lip symmetry is also a limiting factor for this harmonious relationship between teeth and lips. When one of the sides contracts more than the other, there can be no harmony between the incisal line and the lower lip line.

### Connector space (Fig. 2.7)

The point where anterior teeth seem to touch each other is called connector space. There is a difference between connector spaces and contact points: contact points are small areas where the teeth meet; connector spaces are larger and may be defined as areas in which two adjacent teeth seem to touch. The best aesthetic relationship of anterior teeth is the one that follows the 50-40-30 rule of connector spaces.^14^ This rule defines that the connector space between incisors should correspond to 50% of the size of these teeth. Ideal connector space between central and lateral incisor is 40% of the length of the central incisors, and between lateral incisor and canine, 30% of the same reference. Therefore, every time there is no black space or diastema between two teeth, because the space is filled by the gingival papilla, the limits of the connector zone are defined by the papilla tips and the contact points. Therefore, the papillary line and the line of the contact points are the references to determine the connector zone. The design of this zone resembles the shape of a hang glider.

### SMILECURVES TEMPLATE (SCT) (FIG. 3)

**Figure 3:**
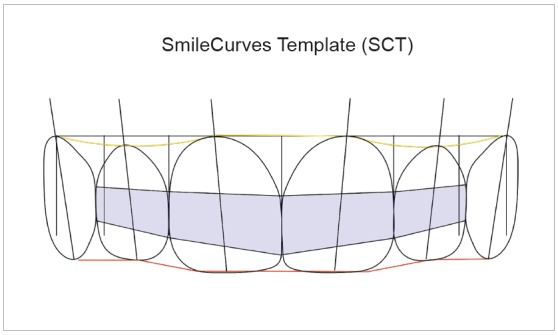
SmileCurves Template (SCT).

The concepts of DDAR and the six horizontal smile lines are the bases of the SmileCurves template, a tool to facilitate the evaluation of dental and oral aesthetics. 

SCT is composed of diagrams that represent the six maxillary anterior teeth, and the principal elements are the central incisors. The ideal proportion between height and width of MCI is about 80%. That is, when width is 8.0 mm, height is 10.0 mm. Based on this, dimensions follow the diagonal proportion of a square (1:1.414), which means that the width of lateral incisors corresponds to 71% of the width of central incisors; and that of the canines, 71% of that of the lateral incisors (regressive proportion). This proportion was chosen because dentists, academics and laypeople prefer it.^6^ The proportion between gingival apical heights follows the principles of DDAR and of the six smile lines. The gingival zeniths of the central incisors and canines have the same height, whereas those of the lateral incisors are a little below (0.6 mm) them. In relation to the incisal line, SCT respects the perspective and consequent parallax effect, thus creating a 0.8-mm step between the incisal edges of central and lateral incisors and positions the incisal and occlusal edges of central incisors and canines on the same perspective, creating an incisal line in the shape of a "soup plate". The connector zone is also part of SCT and provides a notion of the ideal positioning of the papillary lines and the contact points. The drawing of these teeth aims to help in the evaluation and visualization of dental and gingival contours. 

**Figure 4:**
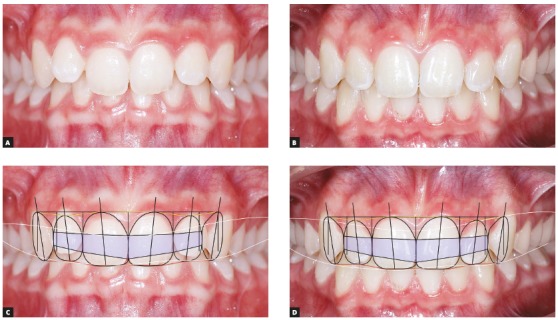
Initial (A) and final (B) intraoral images of patient do not make clear whether deep bite improvement resulted from intrusion of maxillary or mandibular teeth. However, evaluation of smile lines of upper and lower lips, together with SCT, reveals improvement of leveling of maxillary (upper lip line shows that patient had a low smile) and mandibular incisors when comparing images before (C) and after (D) treatment, as there is clear intrusion of mandibular incisors.

**Figure 5:**
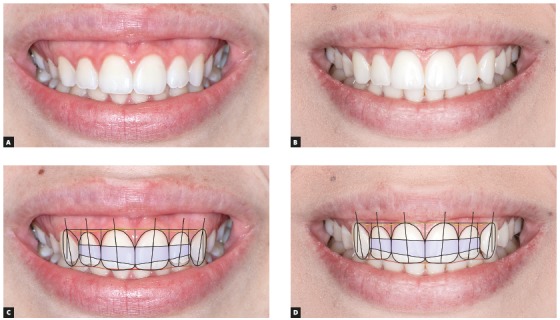
In this case, SCT was used directly over close-up view, and transference of design of lip lines was not necessary. Evaluation of images before (A) and after (B) treatment without SCT does not clearly show whether gummy smile was corrected by increase of clinical crown, orthodontic or surgical intrusion. When template of initial image (C) was positioned, proportion of width to height of incisors was visibly adequate and, therefore, clinical crown increase was not indicated. Final image (D) using SCT shows that gummy smile was corrected by orthodontic intrusion supported by miniplates (posterior region) and mini-implants (anterior region).

**Figure 6:**
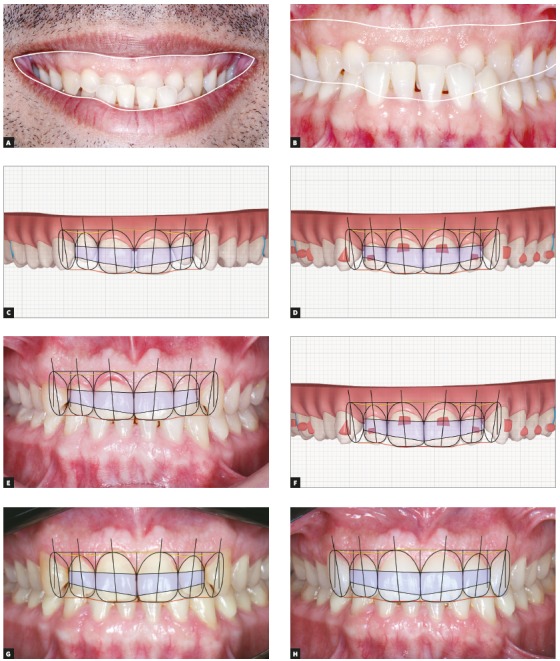
Close-up (A) and frontal (B) intraoral images show that, without full exposure of maxillary teeth, it is not possible to use SCT. However, using Clincheck (Invisalign) image of maxillary arch, SCT can be used on both initial (C) and final (D) images. Comparison of final treatment images (with aligners) (E) using SCT demonstrated Clincheck accuracy (F). Using SCT, periodontal surgery could be directed to increasing clinical crown (G) and final restorations using porcelain laminate veneers (H).

SCT is used with any image software, adapting the template in a PNG file (semi-transparent) over images of JPEG, TIFF, or STL files, or any other image files. SCT may be used in 2D or 3D software, depending only on availability and software adjustment. For that purpose, the width of maxillary central incisors should coincide on frontal smile or intraoral image, and the coincidences, or lack of them, should be evaluated using the DDAR references^1^ and the six horizontal smile lines^2^ described in this text (Figs 4, 5 and 6).

SCT may be useful in several clinical cases. As seen in the examples below, its use was fundamental to achieve better solutions for each type of problem (Figs 4, 5 and 6). In the example in Figure 4, SCT was important for the choice of which teeth, maxillary or mandibular, should undergo intrusion to correct deep overbite. In the clinical case in Figure 5, SCT provided support for the evaluation and choice of treatment to be conducted to correct gummy smile. Finally, the clinical case in Figure 6 shows that SCT may be one more useful tool for 3D planning software programs, such as Clincheck (Invisalign). 

## DISCUSSION

Templates are used to facilitate visualization and interpretation of abstract data, and may turn subjective concepts into objective information.^1^ ^,^ ^2^ ^,^ ^3^ ^,^ ^5^ ^,^ ^6^ SCT performs this function because it facilitates the observation of occlusal and aesthetic characteristics of maxillary anterior teeth in relation to lips, and provides a direct comparison of their components with dental, gingival and labial elements and structures. Although limited by the mutable aspects of a frontal view, due to the parallax effect, it shows all its usefulness, as its characteristics are based on concepts that are similar to others, already seen in the literature.^1^ ^,^ ^2^ ^,^ ^3^ ^,^ ^5^ ^,^ ^6^ ^,^ ^7^ Although the measurements and parameters used in its construction were based on widely used and thoroughly evaluated concepts, SCT might not be adequate for all individuals. Great individual variation is expected. Even so, this tool is useful, because its purpose is not to have all cases fit the structure perfectly. Its use should be an efficient reference for detailed, customized evaluations of correct or incorrect positioning of dental and gingival structures under evaluation. SCT efficiency shall be better analyzed in further studies to appreciate its convenience. However, the clinical use of this tool, when carefully applied, may be useful to support dental planning of aesthetic positioning of maxillary anterior teeth. 

## CONCLUSION

The use of SCT facilitates the visualization of dental and oral aesthetic needs, and its use may both guide treatment options and provide a comparison of outcomes. Moreover, SCT versatility may extend to 2D and 3D software programs, depending exclusively on software availability and adjustment.
